# Understanding the mechanism of acupuncture in acute cerebral infraction through a proteomic analysis: protocol for a prospective randomized controlled trial

**DOI:** 10.3389/fnins.2024.1365598

**Published:** 2024-03-05

**Authors:** Jiangpeng Cao, Yuanhao Du, Xiumei Yin, Na Zheng, Jiawei Han, Linling Chen, Lanyu Jia

**Affiliations:** ^1^National Clinical Research Center for Chinese Medicine Acupuncture and Moxibustion, Tianjin, China; ^2^Graduate School, Tianjin University of Traditional Chinese Medicine, Tianjin, China; ^3^Department of Acupuncture and Moxibustion, First Teaching Hospital of Tianjin University of Traditional Chinese Medicine, Tianjin, China; ^4^Department of Traditional Chinese medicine, Tianjin Huanhu hospital, Tianjin, China; ^5^Department of Traditional Chinese Medicine, First Hospital of Jilin University, Changchun, China; ^6^Department of Traditional Chinese Medicine, Huzhou Central Hospital, Zhejiang, China; ^7^Department of Geriatric Medicine, Tianjin Academy of Traditional Chinese Medicine Affiliated Hospital, Tianjin, China

**Keywords:** acute cerebral infraction, acupuncture, proteomic analysis, randomized controlled trial, protocol

## Abstract

**Background:**

Acute cerebral infarction (ACI), being the predominant form of stroke, presents challenges in terms of the limited effectiveness of various treatments in improving the neurological function. Although acupuncture shows promise in addressing ACI, the availability of high-quality evidence regarding its efficacy, safety, and underlying mechanism remains insufficient. In this study, we design a multicenter, prospective, single-blind, randomized controlled trial with the aim of evaluating the efficacy and safety of acupuncture for ACI, making an attempt to unveil the molecular mechanisms by proteomic.

**Methods:**

A total of 132 patients involving four hospitals will be randomized at a 1:1:1 ratio in the acupuncture group, control group, and sham acupuncture group. All the patients will receive basic treatment, and the patients in the acupuncture and sham acupuncture groups will also receive either acupuncture or sham acupuncture treatment, respectively, at six sessions each week for a 2 weeks period, followed by 3 months of follow-up. The primary outcome will be the change in the National Institute of Health Stroke Scale (NIHSS) scores after treatment. The secondary outcomes will include the Fugl-Meyer Assessment (FMA) scale scores and the Barthel Index (BI). Adverse events that occur during the trial will be documented. To discover differentially expressed proteins (DEPs) and their roles between the ACI subjects and healthy controls, we will also perform 4D-DIA quantitative proteomics analysis, and the DEPs will be confirmed by enzyme-linked immunosorbent assay (ELISA). This study was approved by the institutional review board of the First Teaching Hospital of Tianjin University of Traditional Chinese Medicine (TYLL2023043). Written informed consent from patients is required. This trial is registered in the Chinese Clinical Trial Registry (ChiCTR2300079204). Trial results will be published in a peer-reviewed academic journal.

**Discussion:**

The results of this study will determine the preliminary efficacy and safety of acupuncture in ACI patients and whether the mechanism of this form of non-pharmacologic stimulation is mediated by a novel therapeutic target for neurorehabilitation through our proteomic analysis.

**Clinical trial registration:**

https://www.chictr.org.cn, identifier ChiCTR2300079204.

## 1 Introduction

Acute cerebral infarction (ACI) is the predominant form of stroke, comprising 60–80% of all stroke cases. It is also recognized as the second leading cause of death and the major cause of disability worldwide (Naghavi et al., 2017). The Global Burden of Disease Study has revealed an upward trend in the incidence of ischemic stroke in China, with rates escalating from 117 per 100,000 individuals in 2005 to 145 per 100,000 individuals in 2019 (GBD 2019 Stroke Collaborators, 2021). In addition, the American Heart Association’s report on Heart Disease and Stroke Statistics in 2020 indicates that the prevalence of stroke in the United States was 2.5% in 2016 (Virani et al., 2020). Patients with ACI experience alterations in their lifestyle, which subsequently impacts their overall quality of life (Pan et al., 2008; Wu et al., 2014). These alterations encompass various aspects such as physical wellbeing, psychological state, social interactions, and personal beliefs (Sainsbury and Heatley, 2005). It is widely recognized that neuroprotection holds promise as a therapeutic strategy for individuals affected by ACI. The occurrence and progression of ACI involve multiple pathological and physiological mechanisms, including excitotoxicity, oxidative and nitrosative stress, cellular apoptosis, and inflammation (Lipton, 1999; Chamorro et al., 2016). Acupuncture, a significant component of traditional Chinese medicine, has emerged as a potentially efficacious non-pharmacological intervention for the management of ACI. Acupuncture has exhibited diverse neuroprotective and reparative properties that contribute to its anti-inflammatory (Cao et al., 2021) and anti-apoptotic effects (Tian and Wang, 2019) and its ability to promote angiogenesis (Zhang et al., 2020). The findings from a robust randomized controlled trial involving 862 participants in the early stage of stroke have demonstrated that the addition of acupuncture to standard care leads to a reduction in mortality compared with standard care alone (Zhang et al., 2015a). Numerous reviews have demonstrated the prospective effectiveness and safety of acupuncture in addressing ACI and its subsequent complications. According to a comprehensive examination of literature and perspectives (Qin et al., 2022), it has been observed that acupuncture facilitates the regeneration of nerve cells, thereby promoting the replacement of non-functional cells after a stroke. This process ultimately leads to the restructuring of neural connections and the restoration of functional abilities. Furthermore, a systematic review (Sang et al., 2022) has indicated that acupuncture therapy administered during the sub-acute phase of ischemic stroke may improve language function in individuals with aphasia. Moreover, another systematic review (Wang et al., 2012) has demonstrated that scalp acupuncture exhibits superior efficacy in terms of improving neurological deficit scores and clinical effectiveness compared with western conventional medicine in patients with ACI. Nevertheless, it remains challenging to derive definitive conclusions based on the currently accessible evidence. Further large-scale, well-designed randomized clinical trials on this topic are still warranted (Wong et al., 2012; Zhang et al., 2013a; Li et al., 2014a).

Since “time is brain,” the prompt identification of cerebral ischemia is crucial in the acute phase of onset. Biomarkers serve as objective indicators for evaluating normal or pathological processes, assessing responses to medical interventions, and predicting outcomes (Atkinson et al., 2001). There are already few studies that analyze variations of blood biomarkers after treatment with acupuncture in patients affected by ACI. A recent study (Cui and Jia, 2022) demonstrated that acupuncture in patients with ACI cause an increase in the serum levels of malondialdehyde, superoxide dismutase, and glutathione peroxidase, and another clinical trial (Zhang et al., 2013b) reported a decrease in the levels of serum neuron-specific enolase, soluble protein-100β, and endothelin in ACI patients treated with electroacupuncture. Based on these findings, we hypothesized that alterations in the proteomic profile of blood following acupuncture interventions may potentially mirror neural modifications in individuals diagnosed with ACI.

In light of the ambiguous mechanism and uncertain effectiveness of acupuncture in treating ACI, this randomized clinical trial was devised to assess the safety and efficacy of acupuncture. Additionally, the proteomic analysis will be employed to investigate the potential underlying mechanism. Considering the potential variations in protein expression levels that may arise from acupuncture intervention, the initial set of differentially expressed proteins (DEPs) will be classified based on their associations with specific pathways and then confirmed by the enzyme-linked immunosorbent assay (ELISA) techniques. The validation procedure will be predicated upon the “threshold” of variation of the expression levels, taking into account the findings of previous studies of our team (Du et al., 2011; Li et al., 2014b, 2017, 2022, 2023) and the available relevant literature.

## 2 Methods

### 2.1 Study design

This is a multicenter, prospective, single-blind, randomized controlled trial that will be conducted in four centers in China. The trial design was approved by the Institutional Review Board of the First Teaching Hospital of Tianjin University of Traditional Chinese Medicine (Tianjin, China) and registered at www.chictr.org.cn (ChiCTR2300079204) in December 2023. This study will be conducted in accordance with the latest version of the Declaration of Helsinki, the Consolidated Standards of Reporting Trials (Schulz et al., 2010), and the Standards for Reporting Interventions in Controlled Trials of Acupuncture guidelines (Macpherson et al., 2010) in trial designing and reporting.

### 2.2 Participant recruitment

**Figure 1:**
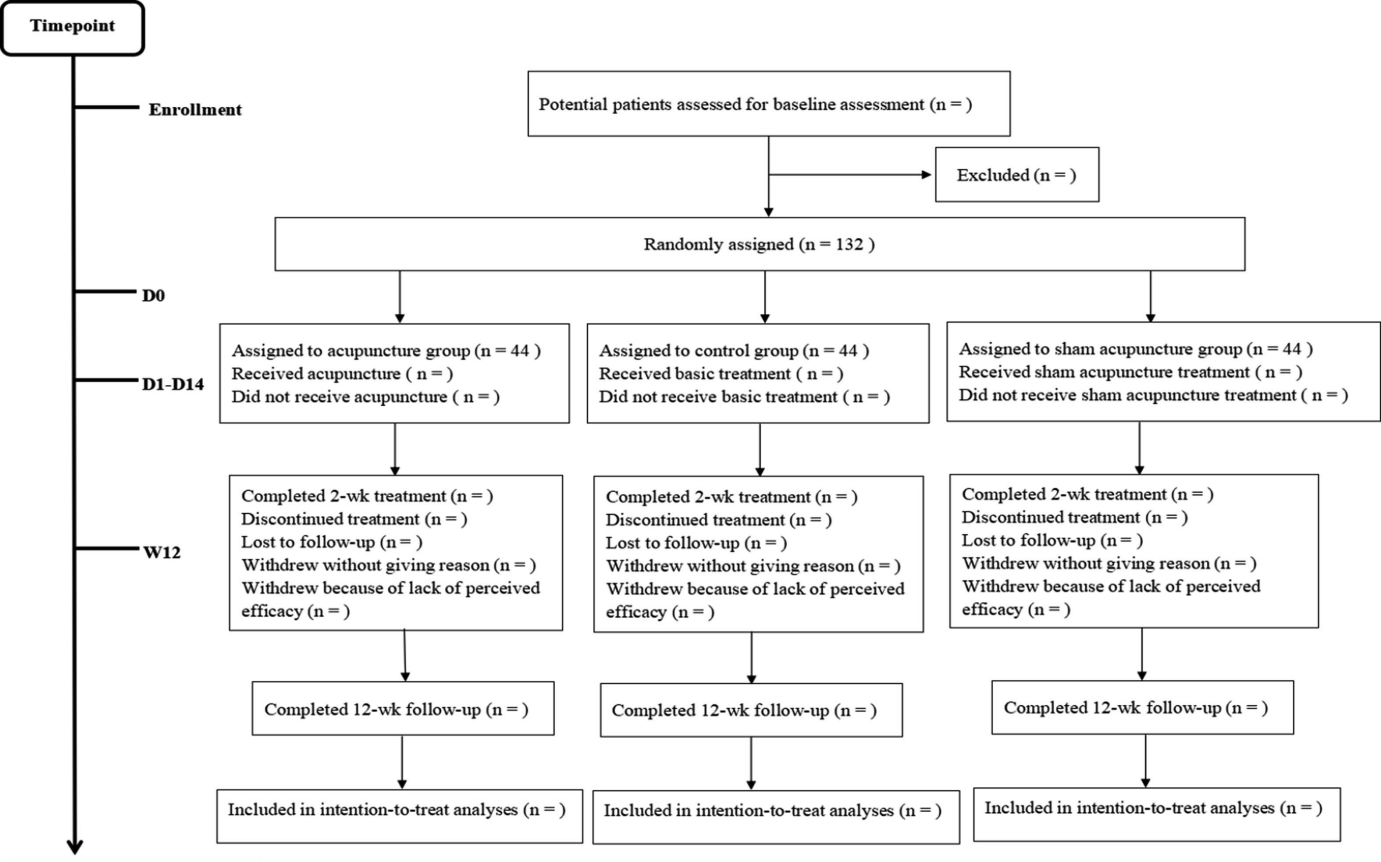
A flowchart of the study.

**Figure 2:**
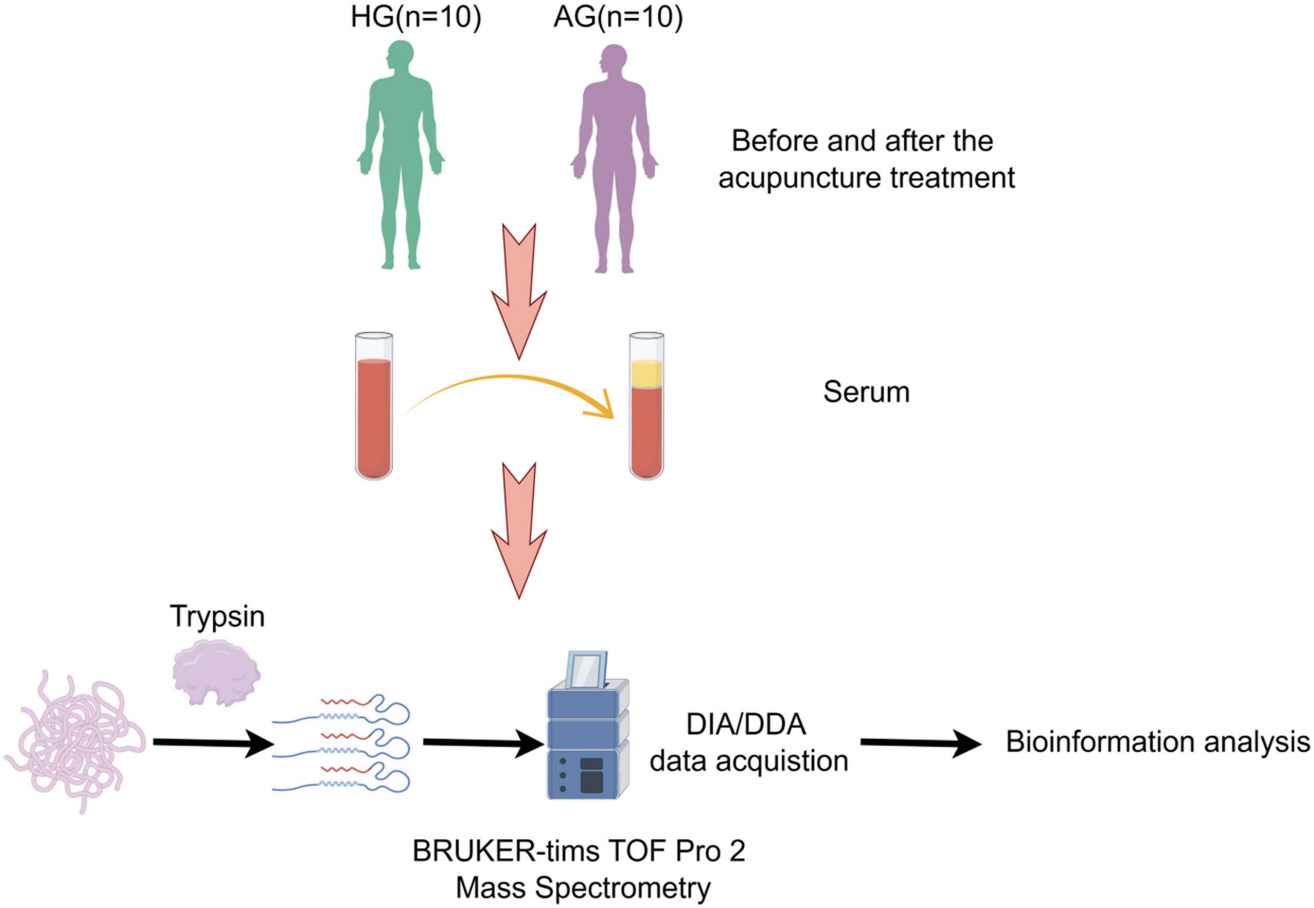
Study design and workflow. AG, acupuncture group; HG, health group; DDA, data-dependent acquisition; DIA, data-independent acquisition.

The eligible consecutive participants will be enrolled in the First Teaching Hospital of Tianjin University of Traditional Chinese Medicine, Tianjin Huanhu Hospital, First Hospital of Jilin University, and Huzhou Central Hospital. This trial will include an enrollment and allocation period of 6 days every week over a 2 weeks intervention period followed by a 3 months follow-up period. The gender- and age-matched healthy people will also be enrolled as the health group that will be analyzed by proteomic analysis investigations. We will obtain the informed consent from each participant or an appointed legal representative if the participant lacks the decision-making capacity. The flowchart of the study is shown in Figure 1, and the mechanism exploration procedure is shown in Figure 2.

#### 2.2.1 The diagnostic criteria

The diagnostic criteria will be designed in accordance with the Chinese guidelines for the diagnosis and treatment of acute ischemic stroke 2018 (Chinese Society of Neurology, & Chinese Stroke Society, 2018).

#### 2.2.2 The inclusion criteria

The inclusion criteria are as follows: (1) individuals who satisfy the established diagnostic criteria for the ailment may be considered for inclusion in the therapeutic intervention; (2) an initial manifestation of the ailment; (3) progression of the ailment within a time frame of 72 h; (4) an age range of 40–75 years with no restrictions based on gender; (5) an NIHSS score ranging from 5 to less than 15; and (6) willingness to participate voluntarily and provision of a duly signed informed consent.

#### 2.2.3 The exclusion criteria

The exclusion criteria are as follows: (1) individuals failing to meet the established inclusion criteria; (2) impairment of the Vesta channel’s functionality resulting from craniocerebral trauma, tumors, and other etiologies; (3) assessment of the impact of acupuncture treatment or other therapeutic medications administered within a 3 months period prior to admission; (4) presence of consciousness disorders, deafness, severe depression, schizophrenia, and inability to cooperate; (5) coexistence of severe primary diseases affecting the cardiovascular, neurological, respiratory, hepatic, and renal systems; (6) severe dermatological conditions or hypersensitivity to the investigational treatment; (7) pregnant, lactating, or expecting women; and (8) patients concurrently participating in other research studies.

### 2.3 Randomization and blinding

The eligible participants will be enrolled and divided randomly in the acupuncture group (received acupuncture), the control group (received no acupuncture), or the sham acupuncture group (received sham acupuncture) at a ratio of 1:1:1 (*n* = 132) using a computer-generated randomization method. Random numbers were put into an opaque envelope and sealed, and the corresponding serial numbers were affixed to the surface of the envelope for random grouping and concealing. The envelopes were opened according to the order in which the subjects were enrolled for grouping and treatment. The patients will be informed whether they will receive acupuncture treatment or not.

The participants in the acupuncture group and sham acupuncture group will be blinded, while those in the control group will not be. Due to the particularity of acupuncture treatment, it is impossible to blind the acupuncturists in this study. All study personnel including the outcome assessors and data analysts will be blinded to the treatment allocation.

### 2.4 Estimation of sample size

In this study, we aim to compare the difference in the NIHSS score between the acupuncture group and sham acupuncture group after the treatment. Sample size calculations were performed for a simple comparison of the two groups. According to a previous study (Sun and Wu, 2019), the NIHSS scores of mean ± SD between the two groups were 6.2 ± 2.1 and 4.9 ± 1.8, respectively, after the treatment. A total of 36 participants per treatment group (108 in total) were required when setting a one-sided test with *α* = 0.025, *β* = 0.20. To compensate for a 20% loss to follow-up, the sample size was increased to 44 participants in each group.

### 2.5 Sample collection and classification

The peripheral blood will be collected in the morning. Blood samples will be taken from the acupuncture and the control groups before and after treatment; then, the participants from both groups will be asked to wait for 30 min after the blood clotted at room temperature; serum will be collected after centrifuging at 3,000 g for 10 min at 4°C and will be stored at −80°C for proteomic analysis and ELISA validation. In total, 10 blood samples will also be taken from 10 gender- and age-matched healthy people for proteomic analysis.

### 2.6 Interventions

All groups will receive the basic treatment of ACI according to the Chinese guidelines for the diagnosis and treatment of acute ischemic stroke 2018, including monitoring and controlling for high blood pressure, sugar metabolic disorder, high cholesterol, and risk factors such as diabetes. The acupuncture and sham acupuncture groups will also receive an additional 30 min of acupuncture therapy as bedside treatment by three acupuncturists who had been trained for at least 3 years and have a master’s degree with more than 5 years of clinical experience, 6 days per week for 2 weeks (12 sessions in total).

#### 2.6.1 Acupuncture points

ACI belongs to the “apoplexy” category of traditional Chinese medicine. Traditional Chinese medicine theory believes that the brain is the capital of the *gods*, and the imbalance of *yin* and *yang* and the disorder of *qi* and *blood* lead to the occurrence of apoplexy. Therefore, we choose *dumai* as the main meridian for the treatment of apoplexy concerning its pathway and indications. *Renzhong* (DU26), *Baihui* (DU20), and *Fengfu* (DU16) are three acupoints that are located on the *dumai* and used commonly for the treatment of apoplexy. In addition, *Jingbi* (Ex-HN-21) is also chosen for improving the motor function of the trouble side. The position of acupoints is in accordance with the name and location of Acupoints (a standard of The People’s Republic of China, GB/T12346-2006, 2006). Details of selected acupoints are shown in Table 1.

**Table 1:** Location of the acupoints.

Points	Location
DU16	On the posterior midline, directly below the external occipital protuberance, in the depression between the origins of the trapezius muscle
DU20	At the junction of a line connecting the apices of the ears and the midline, 5 cun (≈100 mm) from the anterior or 7 cun (≈140 mm) from the posterior hairline
DU26	At the junction of the upper 1/3 and middle 1/3 of the philtrum
Ex-HN-21	1 cun (≈20 mm) superior to the junction of the proximal and middle third of the clavicle

#### 2.6.2 Manipulations

Participants in the acupuncture group will be asked to adopt a supine position, breathe normally, and relax their whole body for 2 min, and then, the acupuncture needle will be swiftly inserted into DU26 toward the nasal septum at a depth of 0.3–0.5 cun (≈6–10 mm) with bird-pecking needling until the eyes become wet or developed tears. After this, DU20 will be punctured at an approximately 15⁰ angle to a depth of 0.8–1.2 cun (≈16–24 mm), with the needle rotated for at least 200 revolutions per minute for 1 min. For the DU16, needles will be punctured to a depth of 0.8–1.0 cun (≈16–20 mm), with the lifting, thrusting, and twirling manipulation to attain deqi (a sensation of soreness, aching, heaviness, swelling, or numbness) (MacPherson and Asghar, 2006; Zhou et al., 2011). Finally, the acupuncture needle will be vertically inserted into Ex-HN-21 at a depth of 0.3–0.5 cun (≈6–10 mm) with the lifting-manipulation on a small scale and then removed the needle if the sensation obtained (an electric sensation from the arm to the finger). Sterile, single-use filiform needles will be used in the treatment, with a length of 25 to 40 mm and a diameter of 0.25 mm, manufactured by Suzhou Medical Appliance in Jiangsu Province of China.

The number of needles and duration of treatment in the sham acupuncture group will identical in the acupuncture group, except for any manipulations (without retaining the needle to activate the *qi*). The sham DU26 point was 1 cun (≈20 mm) lateral to DU26, the sham DU20 point was 1 cun (≈20 mm) lateral to DU20, the sham DU16 point was 1 cun (≈20 mm) lateral to DU16, and the sham Ex-HN-21 point was 1 cun (≈20 mm) lateral to Ex-HN-21.

Participants in the sham acupuncture group will also probably choose the same acupoints as the acupuncture group, and participants will not be encouraged to use other therapies for management of ACI throughout the trial. Details will be documented if other therapies used.

### 2.7 Outcomes and assessment procedures

Three independent research assistants will take charge of their data from baseline to 2 weeks and 3 months of follow-up and for evaluating their outcomes.

#### 2.7.1 Primary outcomes

The primary outcome we chose in this study is the NIHSS score that will be measured before and after treatment to evaluate the neurological deficit. There are 15 items in the scale including consciousness, best gaze, motor arm, facia palsy, motor leg, limb ataxia, sensation ability, dysphagia, and neglect. The score of each item scores range from 0 to 4, and the total scores range from 0 to 42. A higher score indicate a more severe neurological deficit.

#### 2.7.2 Secondary outcomes

Secondary outcomes included the FMA scale scores and BI. The FMA scale comprises assessments of the upper extremity (UE, 66 points) and lower extremity (LE, 34 points). The BI scale includes 10 items: continence of the bowels and bladder, dressing, entering and leaving a toilet, bathing, ascending and descending stairs, etc. We select the FMA scale to evaluate the motor function and BI to evaluate the self-care and mobility before and after treatment. In addition, the modified Rankin Scale (mRS), a scale used to evaluate the state of neurological function recovery in ACI patients, will also be assessed when the follow-up period ended (at 3 months).

#### 2.7.3 Safety assessment

Acupuncture-associated adverse events will be documented by outcome assessors throughout the trial, including bleeding, subcutaneous hemorrhage, serious pain, and fainting. All adverse events will be followed up until the adverse events will be resolved.

#### 2.7.4 Mechanism exploration

##### 2.7.4.1 Proteomic analysis

A quantitative proteomics analysis will be performed based on a 4D-DIA method. The samples were mixed with 1%SDC/100 mM Tris-HCL/10 mM TCEP/40 mM CAA (pH = 8.5) and incubated for 1 h at 60°C for one-step reduction and alkylation. LC–MS/MS data acquisition was carried out on a Q Exactive Plus mass spectrometer coupled with an Easy-nLC 1,200 system. The raw MS data were processed by DIA-NN software (V1.7.16) using the library free method. The general function databases that provide annotations (GO, KEGG, etc.) are used to annotate the identified proteins to understand the functional characteristics of different proteins. Data will be filtered by half the number of actual values in either group and conducted logarithmic transformation and imputation with the random value method. The filled data will be used for subsequent analysis.

##### 2.7.4.2 Protein validation

ELISA kits will be applied to detect differences in the DEP levels of serum in patients in the acupuncture group and the control group before and after treatment. Samples will be diluted appropriately, and the data will be analyzed in duplicate according to the manufacturer’s instructions.

### 2.8 Statistical analyses

Quantitative variables will be presented with mean ± SD. The Shapiro–Wilk test and box plots will be used to assess the homogeneity of the quantitative variables. All continuous and independent variables in the groups will be performed by Student’s *t*-test at baseline. The Mann–Whitney U test will be selected if the variance was not homogeneous between two groups. Stata software will be used to analyze the data according to the intention-to-treat principle, and a two-sided *p*-value less than 0.05 was considered significant.

## 3 Discussion

Cerebral infraction, which is a fundamental manifestation of ischemic stroke, initially results in a transient impairment of tissue function due to insufficient blood supply to the cerebral tissue, but over time, it leads to irreversible damage to neurons and supporting structures. Ischemia triggers a cascade of events, including increased extracellular glutamate concentration, increased intracellular calcium levels, and oxidative stress (Straus et al., 2002). Significant progress has been achieved in combating ACI over the past few decades, resulting in a transformative shift in ACI management. This shift has given rise to the emergence of efficacious thrombolytic and endovascular therapies since 1995. Given the criticality of time in preserving brain function, prompt and accurate diagnosis of ACI within the initial hours of symptom onset is imperative. Regrettably, the accessibility of neuroimaging facilities remains limited in many developing nations, while the time-sensitive nature of revascularization therapies further compounds the challenge.

The incidence of ACI is on the rise as the aging population continues to grow (Ye et al., 2015). The complement system, systemic immune responses, and inflammation play crucial roles in the progression of ischemic lesions and overall outcomes, resulting in permanent disability for 15–30% of stroke patients (Macrez et al., 2011; Zhang et al., 2015b). Therefore, there is an urgent need for effective management and diagnosis of ACI patients.

Biomarkers serve as objective indicators for evaluating physiological or pathological processes, monitoring responses to medical interventions, and predicting outcomes. These indicators encompass various molecules, including proteins, metabolites, lipids, and ribonucleic acids, among others, present in body fluids such as blood, cerebrospinal fluid, and urine. Additionally, biomarkers can also be derived from physical measurements of tissues, such as imaging and electrophysiology. Despite the identification of B-type natriuretic peptide, matrix metalloproteinase-9, D-dimer, and glial fibrillary acidic protein as potential diagnostic biomarkers for stroke within 24 h in a recent systematic review (Misra et al., 2020), the currently available biomarkers lack the desired sensitivity, specificity, rapidity, and precision. Consequently, there is a need to investigate a more accurate and sensitive biomarker to assist in the diagnosis and management of ACI. Significant advancements have been accomplished through the collaborative endeavors of scientists worldwide subsequent to the initiation of the Human Genome Project in the early 1990s. Undoubtedly, proteins serve as the agents responsible for physiological functionality and the tangible manifestation of vital phenomena. Clinical proteomics is a discipline that applies proteomic techniques to clinical diagnosis, treatment, prognosis, and etiology. By combining proteomics technology with clinical epidemiology, medical statistics, bioinformatics, artificial intelligence, molecular biology, and other technologies, we can explore the molecular effects and mechanisms of proteins related to diseases so as to prevent diseases, improve diagnosis and treatment level, accelerate disease improvement, and promote human health. Consequently, the investigation of protein structure and function will directly elucidate the mechanisms underlying alterations in life processes, whether occurring within physiological or pathological contexts.

Over the course of approximately 2,500 years, acupuncture has amassed a wealth of experience and is widely recognized as a valuable therapeutic approach for addressing ACI. Based on the traditional Chinese medicine theory, acupuncture can regulate *qi* and *blood*, warm meridians, and activate collaterals, providing some benefits for promoting the recovery of neurological function in patients with ACI. It is also suggested to select acupuncture for the treatment of ACI during the acute and early recovery phases based on the current evidence. Nevertheless, the ongoing discourse surrounding the methodology of acupuncture clinical research persists due to the challenge of employing universally accepted measures to evaluate its efficacy. In light of the need to evaluate the efficacy and safety of acupuncture for ACI and explore its probable mechanisms through a proteomic approach, we designed this study.

The purpose of this study is to evaluate the efficacy and safety of acupuncture as a therapeutic intervention for ACI and identify the possible mechanisms. Herein, a prospective randomized controlled trial will be conducted to investigate the effectiveness of acupuncture in combating ACI. Then, a 4D-DIA quantitative proteomics analysis will be used to clarify the underlying mechanism of acupuncture. Furthermore, the DEPs will be verified by ELISA. For the acupoint selection, we have chosen DU16, DU20, and DU26 from the *du mai*, of which DU16 and DU20 are the Sea of Marrow according to the Gao Wu command point. DU16 act as a function of nourishing the Sea of Marrow and lightening the *shen* and use calming internal *wind*. DU20 can exert functions of calming *wind* and *shen*, pacifying *yang*, benefiting the *brain* and sensory organs, and nourishing the Sea of Marrow. DU26 is an important emergency point, which, together with DU16, consist of the Sun Si Miao Ghost point, which can revive consciousness, benefit the face and nose, and eliminate *wind*. Ex-HN-21 is the extra point on the head and neck, which is located in the supraclavicular fossa above the brachial plexus and act with the function of paraesthesia and paralysis of the upper extremity. We selected three acupoints located in the brain (DU16, DU20, and DU26) from the *dumai*, known for their ability to awake the brain, calm the *shen*, and alleviate *wind*-induced blockages. Furthermore, Ex-HN-21 was also chosen to improve the motor function of patients with ACI. Consequently, this study was undertaken to furnish evidence regarding the effectiveness of acupuncture intervention at the three acupoints located in the brain (DU16, DU20, and DU26) in ACI patients and investigate its underlying mechanism.

## Ethics statement

The studies involving humans were approved by the Institutional Review Board of the First Teaching Hospital of Tianjin University of Traditional Chinese Medicine. The studies were conducted in accordance with the local legislation and institutional requirements. The participants provided their written informed consent to participate in this study.

## Author contributions

JC: Conceptualization, Writing – original draft, Writing – review & editing. YD: Writing – review & editing. XY: Methodology, Writing – review & editing. NZ: Formal analysis, Writing – review & editing. JH: Formal analysis, Writing – review & editing. LC: Validation, Writing – review & editing. LJ: Software, Writing – review & editing.
